# This outcome feels right! subjective evaluations of coin flip outcomes reflect previously stated preferences

**DOI:** 10.1371/journal.pone.0253751

**Published:** 2021-07-02

**Authors:** Mariela E. Jaffé, Rainer Greifeneder

**Affiliations:** Center for Social Psychology, University of Basel, Basel, Switzerland; Bucharest University of Economic Studies, ROMANIA

## Abstract

When facing a difficult decision, individuals may rely on a coin flip to help them come to a conclusion. In some cases, however, individuals might not adhere to the coin’s outcome, but instead report liking or disliking the coin flip’s outcome, and may use this affective reaction to form their decision. In this manuscript we investigate the affective reaction towards the outcome of a coin flip and determine whether this affective reaction provides valid feedback in regards to individuals’ underlying preferences (Hypothesis 1). We further test whether flipping a coin results in a higher alignment between previous preferences and subsequent decisions (Hypothesis 2). We conducted three studies in the lab and with online samples. Throughout all studies we found support for the notion that the affective reactions regarding the coin flip’s outcome validly reflect previously indicated preferences or attractiveness ratings. Contrary to wide-spread expectations, however, we did not find reliable support for the notion that flipping a coin, compared to a control group, leads to decisions that are more in line with the previously stated preferences.

## Introduction

Coin flips are often considered to be useful decision aids. In some cases, such as when deciding which team should start in a soccer game, the coin flip serves as a fair and quick means to determine the decision. In other cases, however, individuals choose to flip a coin to overcome indecisiveness, especially when difficult decisions have to be made. If one cannot come to a conclusion by other means, flipping a coin may be perceived as a simple and effective means to proceed in the decision-making process. Previous research has shown that about 46% of a German sample from the general public (interested in psychological research) had applied this strategy before [1]. Interestingly, both the survey and anecdotal evidence suggest that individuals do not fully outsource the decision to the coin; instead, they report satisfaction or dissatisfaction with the coin flip’s outcome [1]. Consistent with this affective reaction, they may decide to (not) adhere to the outcome (or flip the coin again, until they like the outcome). Given this experience of liking or disliking, it seems that although preferences were unclear before flipping the coin, individuals have a feeling of which option they prefer after having looked at the coin flip’s outcome. This manuscript investigates the affective reaction towards the outcome of a coin flip. By relying on a context in which we have information about individuals’ previously stated preferences, we can test whether the affective reaction of liking or disliking the outcome of a coin flip can serve as valid information about underlying preferences. Three studies find support for the notion that affective reactions towards the coin flip’s outcome validly reflect previous preferences. Contrary to anecdotal evidence, however, we find no reliable support for the notion that flipping a coin compared to a control group leads to decisions that are more in line with previously stated preferences.

### How can a coin flip elicit affective reactions?

In some situations, individuals who are required to make a decision or want to overcome their indecisiveness might not be able to state their preferences explicitly because they are not aware of them. This can be illustrated with the Iowa Gambling Task [2], in which participants develop a feeling and physiologically measurable reaction towards different decision options, before being able to explicitly voice these (hidden) preferences [2, 3]. But even if individuals are unaware of their preferences, their implicit associations may be informative and predict later decisions [4, 5]. Helping individuals to access these “hidden preferences” may therefore prove an advantageous strategy to overcome indecisiveness.

One strategy to help individuals make decisions could be the use of decision-making aids such as a coin flip. The classic perspective on coin flips suggests that individuals use decision making aids as *deciders*, meaning that the coin flip’s outcome determines the decision, e.g., [6]. In this manuscript, however, we investigate a different notion that is closer to identifying individuals’ “hidden preferences:” the coin acts as a decision aid, which informs (but does not determine) individuals’ decisions. In particular, the coin flip’s outcome elicits an affective reaction, which can then be used as an input in decision making. The coin flip therefore does not decide, but *catalyzes* the decision-making process [7].

Using a (dichotomous) random device such as a coin flip results in receiving a clear suggestion. If the coin acts as a catalyst, this suggestion is not binding, but may nevertheless change downstream processing. In particular, individuals may imagine actually obtaining the coin-suggested option. These concrete mental simulations could then evoke stronger affective reactions [8]. Therefore, imagining obtaining one option over the other may elicit affective reactions, and individuals may feel that they like or dislike the coin flip’s outcome [7]. If the affective reactions are negative, individuals might interpret these reactions as disliking the coin flip’s suggestion. If the affective reactions are positive, individuals might interpret these feelings as liking the coin flip’s suggestion. As a further consequence, they might feel that they know what they prefer, even though they were undecided before [7].

To the extent that affective reactions reflect underlying preferences (and are not driven by situational and irrelevant cues), affective reactions regarding the coin flip’s suggestion could serve as a *valid* input for individuals’ underlying preferences. We therefore hypothesize:

**Hypothesis 1:** Affective reactions in regards to the coin flip’s suggestion validly inform individuals about their underlying preferences. To this end, we test whether the affective reactions towards the coin flip’s outcome are more positive if the coin points to an option previously rated as more attractive, and more negative if the coin points to an option previously rated as less attractive.

### Do affective reactions improve decision quality?

Extant literature suggests that feelings are used in decision-making, and may even constitute a critical ingredient [2, 9–12]. From a philosophical perspective, it has been argued that feelings are the counterpart to rationality, a conflict between “divinity and animality” [13]. The classical view states that feelings can be a disruptive factor in making decisions, but more recent works question this view [14, 15]. Moreover, previous research has suggested that relying on feelings can even improve decision-making [16].

Building on this research and the assumption that the use of affect may increase decision quality, using a coin flip could be beneficial. As highlighted in Hypothesis 1, a coin flip may result in affective reactions such as liking or disliking the outcome. These feelings of liking or disliking the coin flip’s outcome can then be used to make decisions.

This assumption is at the heart of the feeling-as-information account, e.g., [17, 18]. In accordance with the “How do I feel about it” heuristic [19, 20], judgmental targets elicit feelings, and individuals can recruit these elicited affective reactions when evaluating the target [21]. If the targets elicit a positive affective reaction, individuals can interpret that as a positive attitude towards the target. If the target, however, elicits a negative affective reaction, individuals can interpret this as a negative attitude towards the target. Individuals can therefore derive information about their preference and then make a decision in line with this preference.

If a coin flip results in affective reactions that validly reflect individuals’ preferences, one may hypothesize that using coin flips as catalysts may improve decision making. In a setting of preference decisions (i.e., there is no right or wrong), this would signify that using a coin flip results in decisions that are more in line with inherent underlying preferences. We therefore hypothesize:

**Hypothesis 2:** In comparison to a control group, using a coin flip results in higher preference alignment. We test whether using a coin flip results in a stronger alignment between previously stated preferences and subsequent choices.

### General overview of the studies

**Fig 1:**
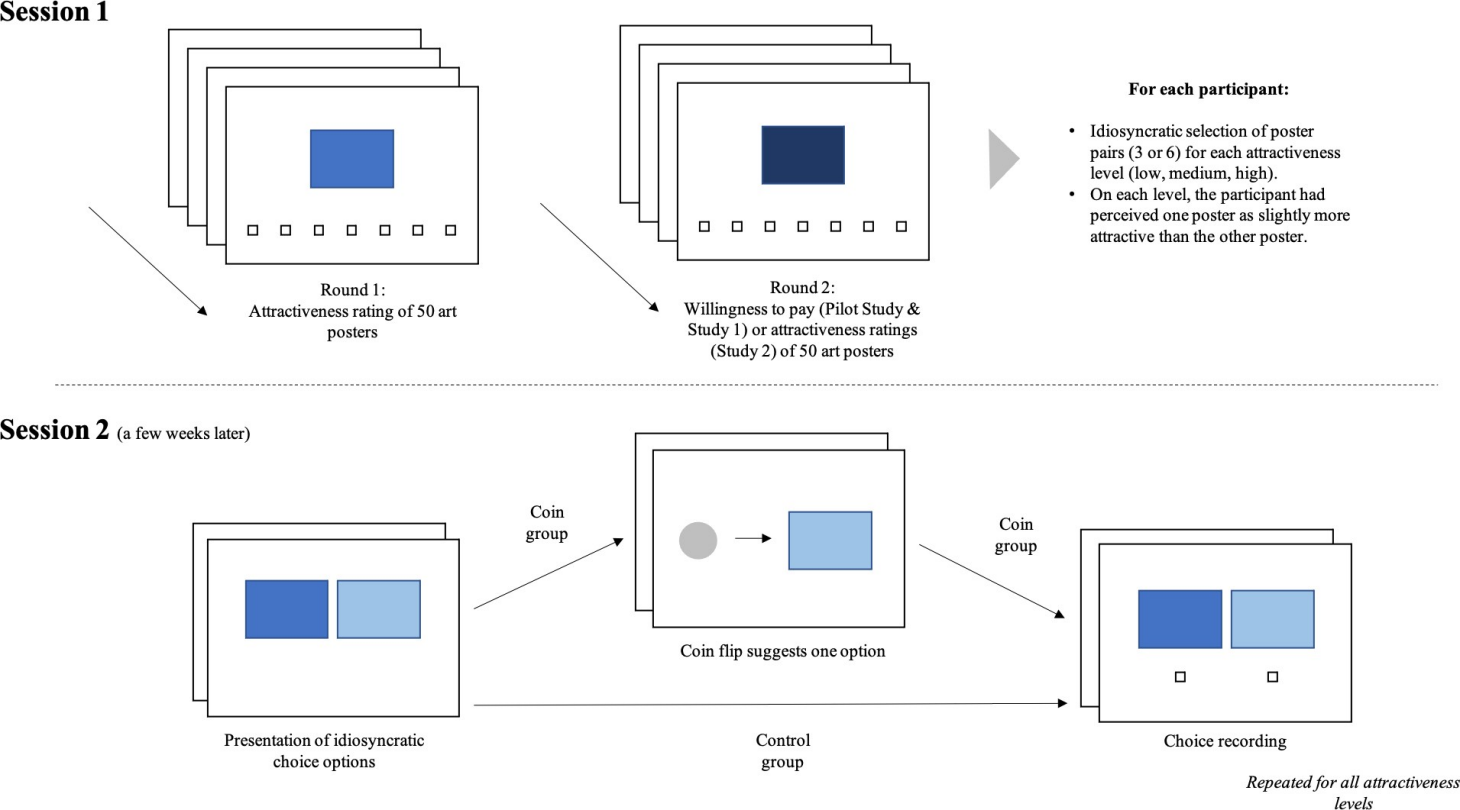
Illustration of procedures used throughout the presented studies.

To investigate how a coin flip impacts individuals’ decision-making processes, we conducted a pilot and two studies. The Institutional Review Board of the University of Basel approved the research (Proposal #001–14). All of the studies consisted of two sessions and were inspired by the research on affective forecasting by Gilbert and Ebert [22, 23]. In the first session we assessed individuals’ attractiveness evaluations of different art posters. These evaluations were then used to create decision scenarios for the second session, conducted a few weeks later. Here, individuals were asked to make a preference decision between two posters, one of which they had previously rated as slightly more attractive than the other. By this means we created a preference decision situation, in which we can test whether the decision option preferred in Session 1 (i.e., the “better” option) stands a higher chance of being chosen in Session 2. To test whether a coin flip facilitates choosing the previously preferred option, half of the participants were asked to flip a coin and to evaluate the outcome before subsequently making their decisions between the two pieces of art (see also Fig 1 for an illustration of the Study procedure).

## Pilot study

We tested the two hypotheses in an experimental setting at a Swiss university. Hypothesis 1 holds that affective reactions in regards to the suggestion of a coin flip in Session 2 are informative in regards to individuals’ preferences assessed in Session 1. For Hypothesis 1, we tested whether the affective reactions towards the coin flip’s outcome are more positive if the coin points to an option previously rated as more attractive, and more negative if the coin points to an option previously rated as less attractive. For Hypothesis 2, we test whether using a coin flip results in a stronger alignment between evaluations in Session 1 and the choice in Session 2.

To measure preferences in Session 1, we assessed individuals’ evaluations of a set of 50 art posters. In Session 2, we then asked individuals to make several pair-wise decisions. In particular, separately for every participant, we created three sets with two options each, one set with posters that the participant previously evaluated as averagely attractive (average ratings), one set where the posters were evaluated as highly attractive (average + 1SD), and a final set in which the posters were evaluated as rather unattractive (average– 1 SD). These sets were constructed in such a way that one poster within a set had previously been rated as slightly more attractive than the other. This setup created a rather difficult task, for several reasons. First, when evaluating 50 pieces of art, most people likely have difficulties remembering the individual pieces and their exact ratings, especially when asked a few weeks later. Second, because within each set, participants preferred one poster only slightly over the other, the two posters within each set were preferred to a very similar extent.

### Methods

#### Participants and design

Session 1 was conducted in the lab as part of a larger set of experiments and was completed by 42 participants (9 male, 33 female, *M*_*age*_ = 23.36, *SD*_*age*_ = 8.15). Participants were asked to provide their email address to be invited to Session 2. Forty participants agreed and provided their email addresses. Session 2 was conducted online and participants received an email with an individual link to the study. Session 2 was completed by thirty-three participants (7 male, 26 female, *M*_*age*_ = 23.85, *SD*_*age*_ = 9.02). Participants received 4 CHF (approximately 4 USD) as compensation for Session 2 and had the chance to win one of the chosen posters.

Due to unknown effect sizes related to our research question, we did not compute an a priori power analysis before conducting the study. We recruited a small convenience sample to investigate potential effect sizes and tendencies in preference decision settings. Due to the small sample size, descriptive and inferential results have to be treated carefully.

The study’s design was a between design with condition (coin versus control) as the independent variable. Within the coin condition, we further manipulated validity of the coin flip’s suggestion (coin suggests more versus less attractive option) as an additional independent variable. Participants made three choices (trials), which differed in regards to the overall attractiveness rated in Session 1 (low, medium, high). Affective reaction to the coin flip (feeling wrong versus right) and alignment with initially indicated preference (henceforth referred to as preference alignment) serve as dependent variables.

#### Materials and procedure

Fig 1 briefly illustrates the procedure used throughout all studies.

*Session 1*. In Session 1 we used a set of 50 classical art prints from different artists. Art prints were downloaded from an online art poster distributor. All participants rated all 50 posters in Session 1 (first with respect to attractiveness, than a second time for willingness to pay; see below).

Participants were welcomed to the lab and informed that they were participating in a study in which they would evaluate art prints. They were then shown 50 different art prints and asked to evaluate how much they liked each print on a scale from -4 (don’t like the art print at all) to +4 (I like the art print very much; coded as 1 to 9). Next, we asked participants for their willingness to pay for the different art prints. Participants received a fictitious budget of 500€ and were asked to indicate their preferences in regards to specific prints. If all prints were equally attractive, they could assign a bid of 10€ to each of the 50 prints. If, however, they perceived the prints as varying in attractiveness, they could also vary their bids. Lastly, participants were asked to provide demographic information, indicate how carefully they had completed the study, and whether there were any reasons not to use their data. Participants then learned that there would be a second part of the study a few weeks later, to which they would be invited if they provided their email-address.

*Session 2*. In preparation for Session 2, we identified five pairs of posters for each individual (two as practice and three as test trials). To select posters for the three test trials, we computed the mean and standard deviation across all attractiveness ratings for each participant. For each participant, we then determined one poster that was rated slightly above the idiosyncratic attractiveness average, and one below (medium trial). We conducted the same approach for the posters high (low) in attractiveness and used the average rating plus (minus) one standard deviation as baseline. In all cases we used a stepwise approach in extending the range of deviance between the poster ratings to find two posters that matched our criteria of being rated on a certain level of attractiveness (medium versus high versus low) while still being at least one rating scale point apart. Furthermore, we checked whether the differences in attractiveness ratings were reflected in an equal (meaning no) or coherent difference (meaning willingness to pay was higher for the poster rated as more attractively) in willingness to pay.

Six to seven weeks after Session 1, participants who had provided their email address were invited to Session 2. Participants received an individual link to start working on the study and were asked to choose between some of the art posters that they had evaluated in Session 1. Participants further learned that they would receive money as compensation, but would also have the chance to win one of the chosen art posters. After providing written informed consent, participants in the coin condition were then introduced to the decision-making aid. They were allowed to try the coin flip up to three times so that they could find out for themselves that the coin produced random outcomes. Coin-participants further learned that in each round, the coin flip would randomly recommend either the left or the right option, depending on the outcome (the assignment of outcome and recommendation could be chosen by participants themselves).

All participants then proceeded to two practice trials. Two posters were presented, one on the left and one on the right, and participants were asked to choose which poster they would prefer to win. Before making this choice, coin-participants saw the coin flip, its recommendation, and were asked to evaluate how wrong or right the coin’s recommendation felt on a 7-point Likert scale (affective reaction; 1 = *wrong*, 7 = *right*).

Next, participants proceeded to the three test trials. Test trial 1 contained the posters that were rated as averagely attractive; Test trial 2 the highly attractive; Test trial 3 the rather unattractive options.

After having completed all trials, participants were asked to indicate the certainty with which they had made each choice. To this end, participants again saw the two practice and the three test trial posters and were asked to indicate how uncertain versus certain they felt when making the respective choice (1 = *very uncertain*, 7 = *very certain*). At the study’s end participants were asked how carefully they had completed the study and whether there were any reasons not to use their data. Participants were thanked for their participation.

### Results

#### Session 1

On average across all posters, participants evaluated the art posters close to the scale mean (*M* = 5.30, *SD* = 0.75). Inspection of the standard deviations regarding attractiveness ratings within each participant further indicates that for all participants some posters were perceived as attractive whereas others were evaluated as unattractive (*M* = 1.96, *SD* = 0.47).

#### Session 2

Table 1 provides descriptive statistics about *affective reactions* following the coin-flip, separately for the three test trials. Results suggest that coin-participants descriptively evaluated the coin more positively when it pointed to the option that they had evaluated as more attractive in Session 1 (Hypothesis 1). Effect sizes differ for the different levels of attractiveness, with larger effect sizes for the medium and low attractiveness trials compared to the high attractiveness trials.

**Table 1:** Affective reactions (means and standard deviations) in the pilot study depending on whether the coin flip suggested the previously more or less attractively rated option.

	Coin points to		
	More Attractive Option	Less Attractive Option	Comparison
Test Trials (Attractiveness Level)	*Mean (SD)*	*Mean (SD)*	*t*-test
Medium	6.14 (1.07)	2.60 (1.96)	*t*(15) = 4.34, *p* = .001, *d* = 2.14
High	5.29 (2.06)	3.50 (1.72)	*t*(15) = 1.95, *p* = .070, *d* = 0.96
Low	5.56 (1.59)	2.38 (1.41)	*t*(15) = 4.34, *p* = .001, *d* = 2.11

*Note*. Higher values indicate more positive reactions. Test trials are ordered according to the arrangement in the study. Independent sample t-tests were computed for every test trial.

With respect to Hypothesis 2, Table 2 provides descriptive statistics about the percentage of participants who chose the option they had rated more positively in Session 1. Results indicate that over 70% of participants chose the previously better rated option throughout all three trials. Descriptively, coin-participants were more likely to choose the better option for the medium and low attractiveness trials; in the high attractiveness trial, however, control-participants were more likely to choose the better option. A generalized linear mixed model (logistic regression model, logit function) using condition as a fixed effect (effect coded with -0.5 for the coin and 0.5 for the control condition), by-participant random intercepts (but not by-test trial random intercepts, as including this random effect resulted in convergence issues), and alignment with the previously better rated option as the dependent variable (1 = *aligned*; 0 = *not aligned*), indicates that condition was not a significant predictor for choice, *b* = -0.53, *SE b* = 0.51, *z* = -1.05, *p* = .296.

**Table 2:** Percentage of preference alignment in the pilot study.

	Percentage of Participants preferring the better rated Option		
	Overall	Coin	Control
Test Trials (Attractiveness Level)	*% (n)*	*% (n)*	*% (n)*
Medium	76 (25)	82 (14)	69 (11)
High	73 (24)	71 (12)	75 (12)
Low	73 (24)	82 (14)	63 (10)

*Note*. Test trials are ordered according to the arrangement in the study.

By exploration, we further investigated whether using the coin flip impacted participants’ decision certainty. To this end we calculated a repeated measures ANOVA with condition as a between factor and trial as a within factor. However, average decision certainty across the three trials did not statistically differ between coin and control group participants, *F*(1, 31) = 0.03, *p* = .857, η^2^_p_ = .00; *M*_*coin*_ = 4.84, *SD* = 1.58; *M*_*control*_ = 4.75, *SD* = 1.34. Furthermore, results indicate no significant main effect of trial, *F*(2, 62) = 2.52, *p* = .089, η^2^_p_ = .08, or interaction effect of trial and condition, *F*(2, 62) = 0.04, *p* = .960, η^2^_p_ = .00. Detailed descriptive results are reported in S1 Table.

### Discussion

The Pilot Study’s results provide preliminary support for Hypothesis 1, namely that affective reactions in regards to the suggestion of a coin flip are consistent with individuals’ previous evaluations. Although we did not hypothesize differential effects for the different attractiveness levels, effect sizes were larger for the medium and low attractiveness level and lower for the high attractiveness level.

In regards to Hypothesis 2, we see that for low and medium attractiveness trials, preference alignment was higher in the coin compared to the control group. For high attractiveness trials, however, the control group showed higher levels of preference alignment. Independent of experimental condition, participants displayed an overall high alignment between their evaluations in Session 1 and their choices in Session 2. We stress that results from the Pilot Study should be treated with caution due to the small sample size. We therefore conducted a second study with the same material but a larger sample to test the hypotheses more thoroughly.

## Study 1

Study 1 closely resembles the Pilot Study and was conducted to replicate previous findings with a larger number of participants and choice trials. In Study 1, participants made eight choices in Session 2 (two practice trials and six test trials). Furthermore, we assessed individuals’ dispositional decision-making preferences (e.g., faith in intuition) and beliefs (e.g., superstition) for exploratory purposes.

### Methods

#### Participants and design

Study 1 was conducted as an online study and participants were recruited via Facebook and the online portal www.forschung-erleben.de, which communicates social psychological research to the German-speaking public. One hundred forty-four participants (42 male, 102 female, *M*_*age*_ = 25.71, *SD*_*age*_ = 5.59) completed Session 1 and participated in a raffle for five vouchers for a large online trader as compensation. Participants were further asked whether they would provide their email address to be invited to the study’s second part. One hundred thirty-four participants agreed and provided their email addresses. Session 2 was conducted about one to two weeks later and participants received an email with an individual link. Session 2 was completed by 114 participants (35 male, 79 female, *M*_*age*_ = 25.96, *SD*_*age*_ = 5.81). All participants were entered into the raffle as compensation for Session 2 and, when interested, participants furthermore had the chance to win one of the posters they had selected.

According to a sensitivity power analysis using G*Power [24], a minimal effect size of *d* = 0.47 could be detected under standard criteria (α = 0.05, one-tailed in a t-test, 1-β = 0.80) with a sample of *n* = 114 participants. This minimal effect size is similar to effects found in previous studies investigating the effect of coin flips on the strength of affective reactions [7]. The effect size for the effect of coin flips on choice behavior is unknown, but likely smaller.

The study’s design was a mixed design with validity of the coin flip’s suggestion as a between subjects factor (coin suggests more versus less attractive option) when testing Hypothesis 1, and condition as a between subjects factor (coin versus control) when testing Hypothesis 2. Participants made six choices (test trials), which differed in regards to the options’ attractiveness (two low, two medium, two high). The trial information was included as a repeated measure. Affective reaction with respect to the coin flip’s outcome (feeling wrong versus right) and alignment with initially indicated preference (short: preference alignment) served as dependent variables.

#### Materials and procedure

Materials and procedure of Study 1 were similar to the Pilot Study except for the changes described below. In Session 2, participants first worked on two practice trials, which included an identical set of art posters for all participants. Next, participants worked on six test trials. The test trials featured posters selected specifically for each participant as a function of their preferences indicated in Session 1. Furthermore, we checked whether the differences in attractiveness ratings were also reflected in an equal or coherent difference in willingness to pay.

In Session 2, at the study’s end, we additionally asked participants to complete the 15-item Faith in Intuition Scale [25], as well as eight items on trait superstition, e.g., “Some things that tend to bring me luck may not be lucky for other people” taken from Trait Superstition and Good-Luck Charms scales from [26, 27]. Furthermore, we added five items to assess individuals’ belief in fate; some items were taken from [27, 28], others were self-developed (e.g., “Fate determines the succession of events in my life”).

### Results

#### Session 1

Looking at participants who provided their email address only, on average, participants evaluated the art posters as attractive (*M* = 5.16, *SD* = 0.77). The average standard deviation indicated that some posters were perceived as more attractive whereas others were evaluated as unattractive (*M* = 2.04, *SD* = 0.48).

Poster selection for Session 2: Based on these ratings, we selected for every participant six posters: two on each level of attractiveness (low, medium, high). For this selection, the same algorithm as in the Pilot Study was used. This algorithm performed well in the large majority of cases. However, for some participants, the algorithm did not yield a unique solution, making further adjustments necessary. In particular, for six trials (three participants), the resulting poster pairs for the medium attractiveness level trials were rated identically. Yet, taking willingness to pay into account differentiated the more attractive from the less attractive option in five out of six trials (for one trial, both attractiveness ratings and willingness to pay were identical, resulting in a trial where no preferred option exists). If missing values occurred for the medium attractiveness level trials, we substituted alternative pictures from the lower rated options or the higher rated options, accordingly. Note that we do not know which participants dropped out between Sessions 1 and 2, so that the above described adjustments may or may not have influenced the set of posters rated in Session 2.

For very few cases (n = 8), the algorithm did not result in enough options for the low or high attractiveness trials. In this case, the participant was not invited to participate in Session 2.

Looking at the selected options separated by attractiveness level, mean difference scores between idiosyncratic ratings across participants from Session 1 are *M*_*Medium1*_ = 1.02 (*SD* = 0.25), *M*_*Medium2*_ = 1.17 (*SD* = 0.68), *M*_*Low1*_ = 1.01 (SD = 0.09), *M*_*Low2*_ = 1.02 (SD = 0.12), *M*_*High1*_ = 1.00 (SD = 0.00), and *M*_*High2*_ = 1.02 (SD = 0.17).

#### Session 2

We calculated a linear mixed model using suggestion validity (whether the coin points to the *more attractive* = 0.5 or *less attractive* = -0.5 option) as a fixed effect and by-participants and by-test trials random intercepts. *Affective reaction* in regards to the coin flip served as the dependent variable. Results indicate that suggestion validity was a powerful predictor for affective reactions, *b* = 1.60, *SE b* = 0.19, *t*(331.51) = 8.41, *p* < .001. In particular, coin-participants evaluated the coin more positively when it pointed to the option that they had evaluated as more attractive in Session 1 (see Table 3).

**Table 3:** Affective reactions (means and standard deviations) in Study 1 depending on whether the coin flip suggested the previously more or less attractively rated option.

	Coin points to	
	More Attractive Option	Less Attractive Option
Test Trials (Attractiveness Level)	*Mean (SD)*	*Mean (SD)*
Medium 1	5.32 (1.65)	3.26 (2.16)
Medium 2	4.73 (1.70)	3.54 (2.23)
Low 1	4.94 (1.63)	4.04 (1.72)
Low 2	5.03 (1.36)	2.95 (1.88)
High 1	5.19 (1.90)	4.10 (1.72)
High 2	5.76 (1.70)	3.33 (1.86)

*Note*. Higher values indicate more positive reactions. Test trials are ordered according to the arrangement in the study.

Table 4 provides an overview of the results in regards to Hypothesis 2 and indicates that in general about 73% of participants chose the more attractive option throughout all six trials. Coin-participants outperformed control-participants in three trials; and control-participants outperformed coin-participants in three trials, resulting in a stalemate. Consistent with this description, a generalized linear mixed model (logistic regression model, logit function) using condition as a fixed effect (effect coded with -0.5 for the coin and 0.5 for the control condition, by-participants and by-test trials random intercepts, and alignment with the previously better rated option as the dependent variable (1 = *aligned*; 0 = *not aligned*), indicated that condition was not a significant predictor for choice, *b* = 0.09, *SE b* = 0.19, *z* = 0.48, *p* = .633.

**Table 4:** Percentage of preference alignment in Study 1.

	Percentage of Participants preferring the better rated Option		
	Overall	Coin	Control
Test Trials (Attractiveness Level)	*% (n)*	*% (n)*	*% (n)*
Medium 1	73 (83)	70 (40)	75 (43)
Medium 2	63 (72)	60 (34)	67 (38)
Low 1	68 (77)	70 (40)	65 (37)
Low 2	75 (85)	79 (45)	70 (40)
High 1	77 (88)	70 (40)	84 (48)
High 2	82 (93)	83 (47)	81 (46)

*Note*. Test trials are ordered according to the arrangement in the study.

In an exploratory fashion, we also investigated whether coin-participants were eventually more certain in their preference decisions compared to control-participants (see S1 Table for descriptive results). To this end we calculated a repeated measures ANOVA with condition as a between factor and trial as a within factor. Results indicate no significant effect of condition, *F*(1, 112) = 1.82, *p* = .180, η^2^_p_ = .02, or interaction of trial and condition, *F*(4.37, 489.57) = 0.93, *p* = .450, η^2^_p_ = .01. Average levels of certainty however differed across trials, *F*(4.37, 489.57) = 6.24, *p* < .001, η^2^_p_ = .05.

### Discussion

The results from Study 1 replicate the preliminary findings from the Pilot Study. Participants’ affective reactions towards the outcome of the coin flip in Session 2 reliably reflected initial preferences in Session 1. In particular, if the coin pointed to the option previously rated as more attractive, participants reported a more positive feeling towards the outcome. In contrast, if the coin suggested the option previously rated as less attractive, participants were more likely to report a negative feeling towards the coin. Results provide support for Hypothesis 1. Different from the Pilot Study, visual inspection of the effects across attractiveness levels suggests that effects were present across all levels, yet smaller in magnitude and less variable across attractiveness levels.

Hypothesis 2 states that the participants’ feeling response translates to a higher preference alignment when looking at decisions in the coin compared to the control group. Overall, the observed data is not in support of Hypothesis 2 (for some trials, a trend in favor of Hypothesis 2 is present; for other trials not).

The fact that coin-participants did not outperform control-participants captured our attention. On the one hand, coin-participants had valid affective reactions following the coin flip’s suggestion, meaning that they could correctly evaluate the coin’s outcome as right when it pointed to the more attractive option, and as wrong when it pointed to the less attractive option. On the other hand, this affective reaction did not seem to lead to an advantage regarding choice, as coin-participants were not more likely to reliably choose the option they rated as more attractive in Session 1.

One reason for this unexpected result may be that by asking participants about their feelings regarding the coin flip, we made other cognitions salient, too, thereby decreasing the chance that participants would use their affective reactions when making subsequent decisions. Such a paradoxical effect would be in line with findings by Wilson and colleagues, that individuals who were not asked to provide reasons but simply trusted their intuition showed choice behavior that was more aligned with expert opinions [29] and showed higher levels of choice satisfaction [30]. To test this ex post consideration, we exploratorily tested whether faith in intuition moderates the effect of condition on preference alignment, but did not find any main or interaction effects, all *p*s > .374.

Another reason for this unexpected result may be that participants were generally well above chance level when making preference decisions, thus making it very hard for the coin to further increase the preference alignment level. This overall high preference alignment poses another challenge, as one may entertain the speculation that participants simply remembered which poster they had evaluated as more attractive in Session 1. Although accurate memory appears highly unlikely (participants had rated 50 posters in Session 1; we selected posters that were rated very similarly in Session 1; Session 2 was assessed one to two weeks after Session 1), it cannot be ruled out, for instance because we had used paintings with concrete motives (e.g., the Virgin and Child with Saint Anne by Leonardo da Vinci). To address this concern and to provide an additional test of our hypotheses, we conducted another study in which we employed abstract art pieces created by less well-known artists only, and prolonged the interval between Sessions 1 and 2. With the help of this new setup we created conditions that further minimize the chances for accurate memory.

## Study 2

### Methods

#### Participants and design

Study 2 was conducted as a combined lab and online study, where Session 1 was carried out via tablets on the university campus and Session 2 online. One hundred and thirty-three participants (44 male, 89 female, *M*_*age*_ = 23.63, *SD*_*age*_ = 4.06) completed Session 1 of Study 2 and received course credit and candy as compensation. They were further asked whether they would provide their email address to be invited to Session 2. One hundred participants agreed and provided their email addresses. Session 2 was conducted five to six weeks later and participants received an email with an individual link to start the study. Sixty-three participants provided choice data in Session 2 (14 male, 45 female, 4 missing, *M*_*age*_ = 23.44, *SD*_*age*_ = 2.95). All Session 2 participants were entered into a raffle for online trader vouchers as compensation.

According to a sensitivity power analysis computed with G*Power [24], a minimal effect size of *d* = 0.66 could be detected under standard criteria (α = 0.05, one-tailed t-test, 1-β = 0.80) with a sample of *n* = 63 participants. To be able to analyze Hypothesis 1 with enough power, two-thirds of the participants were randomly assigned to the coin group (*n* = 41), whereas only one-third was assigned to the control group (*n* = 22).

The design of the study was a mixed design with validity of the coin flip’s suggestion as a between subjects factor (coin suggests more attractive versus less attractive option) when testing Hypothesis 1, and condition as a between subjects factor (coin versus control) when testing Hypothesis 2. Participants made six choices (trials), which differed in option attractiveness (two low, two medium, two high). The trial information was included as a repeated measure. Affective reaction to the coin flip (feeling wrong versus right) and alignment with initially indicated preference served as dependent variables.

#### Materials and procedure

Materials and procedure of Study 2 were identical to Study 1, except for the changes noted here. First, we presented abstract art. For the attractiveness ratings in Session 1 we asked participants to rate how much they liked each piece of art on a scale from -5 (don’t like the art print at all) to +5 (I like the art print very much; coded as 1 to 11). Instead of subsequently asking for their willingness to pay, we again showed all posters, and asked participants to evaluate how much they liked each piece of art a second time to gauge the preference stability and increase the reliability of our measure. In both rounds of attractiveness ratings, if participants had accidently forgotten to rate a print, we displayed the option again and asked for a spontaneous rating. However, only the initial ratings (without reminder) were used to select options for Session 2.

In Session 2, we added two further questions. We asked participants how dissatisfied or satisfied they were regarding their decision (1 = *very dissatisfied*, 7 = *very satisfied*) and how difficult or easy it was for them to make the decision (1 = *very difficult*, 7 = *very easy*). Furthermore, for each choice item, we asked participants how attractive the respective piece of art was when presented to them in Session 1 (-5 = *not at all*, +5 = *very much*), henceforth referred to as recalled attractiveness.

### Results

#### Session 1

Looking at participants who provided their email address only, on average, participants evaluated the art posters as attractive (*M* = 5.34, *SD* = 1.23). The average standard deviation indicates that some posters were perceived as more attractive whereas others were evaluated as less attractive (*M* = 2.56, *SD* = 0.58). By tendency, both the grand mean and standard deviation are higher than in Study 1.

Poster selection for Session 2: Based on these ratings, we selected for every participant six posters: two on each level of attractiveness (low, medium, high). For this selection, the same algorithm was used as before. This algorithm performed well in the vast majority of cases. However, for some participants, the algorithm did not yield a unique solution, making further adjustments necessary.

For three participants, the medium or high attractiveness level trials (total 7 trials across the three participants) were rated identically in the first round, but attractiveness ratings in the second round differentiated the more attractive from the less attractive option and were thus used to build trials. Unfortunately, for four participants, the selection process resulted in a wrong compilation of posters in one trial in either the low or the medium attractiveness level. Again, we note that we do not know which participants dropped out between Sessions 1 and 2, so that the above described adjustments may or may not have influenced the set of posters rated in Session 2. Finally, for 15 participants, we substituted alternative pictures to be able to show all participants the same number of trials, even if the algorithm did not result in the selection of sufficient options. The data of these artificially constructed trials were omitted in the data analysis in Session 2.

Looking at the selected options separated by attractiveness level, mean difference scores between idiosyncratic ratings across participants from Session 1 are *M*_*Medium1*_ = 1.01 (*SD* = 0.57), *M*_*Medium2*_ = 1.09 (*SD* = 0.61), *M*_*Low1*_ = 1.01 (SD = 0.10), *M*_*Low2*_ = 0.92 (SD = 0.68), *M*_*High1*_ = 1.02 (SD = 0.20), and *M*_*High2*_ = 1.02 (SD = 0.21).

#### Session 2

To test whether the difference in affective reactions was associated with whether the coin pointed to the more attractive or less attractive option, we calculated a linear mixed model using suggestion validity (whether the coin points to the *more attractive* = 0.5 or *less attractive* = -0.5 option) as a fixed effect and by-participant random intercepts. *Affective* reaction in regards to the coin flip served as the dependent variable. Results from the regression model indicate that suggestion validity was a significant predictor for affective reactions indicated by participants, *b* = 1.38, *SE b* = 0.24, *t*(224.00) = 5.84, *p* < .001. Consistent with Hypothesis 1, these findings suggest that coin-participants evaluated the coin better when it pointed to the option that they had evaluated as more attractive in Session 1. Note that even when computing the least complex model reported above, a warning message indicated singular fit, meaning that inferential statistics need to be treated with caution. When using individual t-tests for every trial, we find significant differences for four out of six trials, see Table 5.

**Table 5:** Affective reactions (means and standard deviations) in Study 2 depending on whether the coin flip suggested the previously more or less attractively rated option.

	Coin points to		
	More Attractive Option	Less Attractive Option	Comparison
Test Trials (Attractiveness Level)	*Mean (SD)*	*Mean (SD)*	*t*-test
Medium 1	5.00 (1.75)	3.78 (1.77)	*t*(36) = 2.14, *p* = .039, *d* = 0.70
Medium 2	4.47 (2.12)	3.45 (1.79)	*t*(35) = 1.59, *p* = .122, *d* = 0.52
Low 1	5.41 (1.66)	3.40 (1.57)	*t*(35) = 3.78, *p* = .001, *d* = 1.25
Low 2	4.76 (1.79)	4.44 (1.86)	*t*(35) = 0.54, *p* = .594, *d* = 0.18
High 1	5.00 (1.79)	3.47 (1.84)	*t*(38) = 2.66, *p* = .011, *d* = 0.84
High 2	5.35 (1.79)	3.29 (1.69)	*t*(35) = 3.58, *p* = .001, *d* = 1.18

*Note*. Higher values indicate more positive reactions. Test trials are ordered according to the arrangement in the study. T-tests are independent sample tests computed for every test trial.

Table 6 provides an overview of the results in regards to Hypothesis 2 and indicates that averaged across trials, over 60% of participants chose the more attractive option. To test Hypothesis 2, we calculated a generalized linear mixed model (logistic regression model, logit function) using condition as a fixed effect (coded -0.5 for the coin and 0.5 as the control condition) and by-participant random intercepts. Alignment with the more attractive option served as the dependent variable (1 = *aligned*; 0 = *not aligned*). Results from the regression model indicate that condition is not a significant predictor for choice, *b* = -0.19, *SE b* = 0.23, *z* = -0.82, *p* = .415. However, even when computing the least complex model, a warning message indicated singular fit, and inferential statistics therefore need to be treated with caution. When computing a Chi-Squared Test for each type of trial, we do not find significant differences (χ^2^(1) < 2.52, *p* > .112), except for the trial High 2, where coin-participants showed higher preference alignment than control-participants, χ^2^(1) = 6.33, *p* = .012.

**Table 6:** Percentage of preference alignment in Study 2.

	Percentage of Participants preferring the better rated Option		
	Overall	Coin	Control
Type of Trial (Attractiveness Level)	*% (n)*	*% (n)*	*% (n)*
Medium 1	59 (35)	58 (22)	62 (13)
Medium 2	59 (32)	60 (22)	59 (10)
Low 1	64 (38)	68 (25)	59 (13)
Low 2	70 (41)	62 (23)	82 (18)
High 1	69 (42)	73 (29)	62 (13)
High 2	67 (37)	78 (29)	44 (8)

*Note*. Test trials are ordered according to the arrangement in the study.

Furthermore, we investigated whether coin-participants were more certain in their preference decisions, more satisfied with them, or found them easier to make compared to control-participants. To this end we calculated a repeated measures ANOVA with condition as a between factor and trial as a within factor. Results indicate no significant effect of condition on either dependent variable, all *F*s < 1. Detailed descriptive results can be found in S1 Table.

Finally, we analyzed participants’ recalled attractiveness. We subtracted recalled attractiveness for the art poster rated as less attractive in Session 1 from recalled attractiveness for the art poster rated as more attractive in Session 1. Values greater than zero indicate correct memory tendencies. Looking at descriptive tendencies only, coin-participants’ memory seems accurate at all attractiveness levels, Medium 1: *M* = 0.89, *SD* = 3.05; Medium 2: *M* = 1.46, *SD* = 3.05; Low 1: *M* = 0.66, *SD* = 3.11; Low 2: *M* = 0.60, *SD* = 2.89; High 1: *M* = 1.26, *SD* = 3.19; High 2: *M* = 1.49, *SD* = 2.12. Control-participants’ participants memory seems also largely correct, but not on all attractiveness levels, Medium 1: *M* = 1.29, *SD* = 3.02; Medium 2: *M* = -0.06, *SD* = 2.88; Low 1: *M* = 0.59, *SD* = 3.26; Low 2: *M* = 1.82, *SD* = 2.54; High 1: *M* = 0.38, *SD* = 1.96; High 2: *M* = 0.50, *SD* = 3.24. Averaged across all attractiveness levels, all difference scores were greater than zero; coin-participants: *M* = 1.16, *SD* = 1.51; *t*(37) = 4.73, *p* < .001, *d* = 0.77; control-participants: *M* = 0.80, *SD* = 1.39; *t*(21) = 2.71, *p* = .013, *d* = 0.58. Conditions did not differ, *t*(58) = 0.91, *p* = .368, *d* = 0.24.

### Discussion

Results from Study 2 replicate the findings from previous studies. Again, participants’ affective reactions towards the coin flip outcome reflect previous preferences very well: If the coin points to the more attractive option, participants report a more positive affective reaction towards the outcome; if the coin suggests the less attractive option, participants are more likely to report a negative affective reaction towards the coin. When looking at the different attractiveness levels in an exploratory fashion, we see that effects were again present across all levels, and while the effects varied in strengths across attractiveness levels, there seems to be no consistent pattern. Overall, results provide support for Hypothesis 1.

Again, however, the results also indicate that this valid affective reaction does not translate into decision making that is more in line with previously indicated preferences (Hypothesis 2). Participants in general were above chance level when making a preference decision, but using a coin did not increase this number above the preference alignment level in the control group.

Study 2 also asked participants in Session 2 to recall the attractiveness they had experienced in Session 1. By means of this recalled attractiveness, we wished to gauge how well participants recall their initial attractiveness ratings. Results suggest that participants’ recall is above chance and does not differ between conditions. However, in retrospect, this measure’s reliability is unclear. It is possible that the measure indeed reflects recalled attractiveness as intended; however, it is also possible that participants constructed their response ad hoc, for instance, consistent with the choices they had just made. This is because recalled attractiveness was assessed after choices had been made. Future research targeting memory accuracy is well advised to use other methodological approaches.

## General discussion

In this manuscript we investigate the affective reaction towards the outcome of a coin flip and determine whether this affective reaction provides valid feedback on individuals’ underlying preferences. We conducted three studies in the lab and with online samples. In all studies we assessed individuals’ preferences regarding art posters in a first session and created idiosyncratic choice situations for the second session. The Pilot Study and Study 1 relied on rather well-known classical art posters (e.g., by artists such as Claude Monet or Leonardo da Vinci), while Study 2 relied on work by unknown artists, for which recognition should be lower.

In Session 2, participants were asked to choose between two art posters, one of which they had previously rated as slightly more positive than the other. Half of the participants flipped a coin before making the decision, whereas the other half did not (control group). We analyzed coin-participants’ affective reaction to the coin flip (feeling wrong versus right) and for all participants the alignment of their chosen art print with an initially indicated preference for this print over the other (short: preference alignment). Throughout all studies we find support for the notion that the affective reactions regarding the coin flip’s outcome validly reflect previously indicated preferences (Hypothesis 1), meaning that when the coin pointed to the previously more (less) attractive poster, participants indicated a more positive (negative) feeling to its outcome. Contrary to wide-spread anecdotal evidence, however, we find no support for the notion that flipping a coin compared to a control group leads to decisions that are more in line with previously stated preferences (Hypothesis 2).

Different explanations may exist for the relatively high levels of preference and choice alignment across all participants. First, one could argue that participants recalled their Session 1 attractiveness ratings when choosing in Session 2. However, it should be kept in mind that participants rated the attractiveness of 50 art posters in Session 1, while choosing only from a subset in Session 2. Moreover, the study procedure was designed to keep differences within in each Session 2 choice pair minimal. While one poster was previously rated as more attractive than the other, this difference generally amounted to just one point on a 9-point (Pilot Study and Study 1) or 11-point (Study 2) Likert scale. Finally, Sessions 1 and 2 were assessed at least one to two weeks apart. Together these measures render recall of previously indicated preferences an unlikely explanation for the high level of observed alignment. It should be noted that the recalled attractiveness data and ratings of difficulty (see S1 Table) in Study 2 may be less reliable than initially intended given that they were assessed after choices had been made.

A second explanation may hold that preferences are stable so that it is no surprise that attractiveness ratings and choice behavior align even across several weeks. While this may potentially account for the pieces of well-known artists in the Pilot Study and Study 1, it seems less likely for the abstract pieces of unknown artists in Study 2.

These first two explanations for the high alignment between preferences and choices may also dampen the excitement about the finding that affective reactions to the coin validly reflected initial preferences. Indeed, if participants recalled their previous ratings and/or preferences were entirely stable, it would not be surprising that participants correctly indicate that the coin felt more right (wrong) when pointing to the previously better (worse) rated option. Being transparent about this possibility is important as it allows us to provide an outlook on how future research can better test the two hypotheses. However, we again highlight that this possibility appears rather unlikely for the reasons stated above. Rather, we turn our attention to at least two more potential explanations for the high alignment between preferences and choices:

A third explanation for the high levels of alignment holds that *all* participants relied on affective reactions when indicating attractiveness in Session 1 and when making choices in Session 2. Previous research suggests that flipping a coin results in stronger affective reactions [7]. While Hypothesis 2 followed this earlier evidence, it is possible that in the context of art, all participants have strong affective experiences, creating a ceiling effect. Unfortunately, an empirical test is forestalled, as we assessed affective reactions only in response to the coin flip and thus lack data on affect for the control group.

A final explanation is closely associated with the third one, but instead of focusing on the strength of affective experiences, it focuses on the *reliance* on feelings. On the one hand, one could argue that *all* participants relied on feelings when evaluating attractiveness and when choosing, thus creating a ceiling effect; presumably the coin flip could not grant a cutting edge over this high baseline. On the other hand, one could argue that coin-participants did not rely on affect as much as they could, which is consistent with the exploratory analysis reported in Study 2. This is further consistent with literature suggesting that individuals are very reluctant to use a coin flip to make decisions [6, 31]. The presented studies in this manuscript forced individuals to use a coin flip to make a decision, but given the strong emphasis on reason-based decision making in the western world [31, 32], participants might have elected to discard the elicited feelings so as not to appear unreasonable.

### Implications

Throughout three studies we show that affective reactions correctly reflect past preferences. These results may explain why anecdotal evidence abounds that individuals know and use the strategy of asking a random decision-making aid for support while still deciding independently: the coin flip strengthens feelings (e.g., satisfaction versus dissatisfaction) that may or may not be used, but could be found helpful, when making a decision, see also [7].

Going beyond affective reactions, we do not find support that a coin flip results in a higher proportion of decisions that are in line with previously stated preferences in the context of art posters. Perhaps this is because a larger sample size would have been needed to detect small effects; or because participants had access to previous preferences; or because participants may have been hesitant to use the coin flip strategy as a way to investigate and use felt reactions (see discussion above).

Even if the coin flip did not result in a stronger alignment of art preferences and art decisions, it may have afforded a change in perspective by providing individuals with felt input to the decision-making process. This change in perspective is not necessarily limited to experimental settings, where participants are asked to use the coin as a decision-making aid. Instead, the coin likely provides felt input when individuals proactively and voluntarily choose to rely on the coin flip. As a result, flipping a coin seems to be a promising strategy especially when individuals face difficult decisions between two options (if more than two options are available, the situation may be trickier and the coin flip perhaps less helpful).

### Limitations and future research

One limitation pertains to the fact that while great care was taken to ensure that within each choice pair, one poster is only slightly better than the other, we did not assess a manipulation check for explicit confirmation. Future research could improve the studies’ setup by implementing checks to assess whether participants have strong or weak preferences for one or the other option, as presumably, affective reaction may inform decisions more strongly when no strong preferences are present. Moreover, the study design could be further improved, for example, by using stimuli that are less recognizable and that are generally not evaluated and chosen based on feelings (e.g., utilitarian vs. hedonic products, see [33]).

We assessed affective reactions in regards to the coin flip suggestion to investigate individuals’ reaction to the coin flip. Interestingly, other research using coin flips suggests that individuals’ preferences can be detected by looking at which option participants assign to heads (the prominent label) versus tails [34]. Indeed, in a set of studies, participants were more likely (compared to chance level) to assign the preferred option to heads (or other prominent labels) [34]. Throughout our studies, we randomly assigned options to heads versus tails, but future research could ask participants to make assignments, and then test whether preferences (both strong and weak) may be revealed from these choices.

Furthermore, future research could rely on settings that may result in less skepticism towards the coin flip. One could try, for instance, to reduce individuals’ felt accountability for the decision [35] or to offer participants the use of the coin flip as an option and not mandatory procedure. Another option could be to offer individuals different random decision-making aids, such as a die or a counting out rhyme. As long as the device provides a random and clear-cut suggestion, we would assume that feelings are strengthened. All of these changes could result in individuals being more open to using their felt reactions when making decisions.

Finally, to the extent that effect sizes are smaller than expected, increasing the sample size will be necessary to detect presumably existing associations. For instance, a stronger sample size may show that feelings elicited by the coin can translate into advantageous decision making in the context of art posters.

## Conclusion

When facing a difficult decision, individuals may rely on a coin flip to help them come to a conclusion. For situations in which individuals might decide not to adhere to the coin’s outcome, but instead act upon their affective reaction of liking or disliking the outcome, this manuscript indicates that affective reactions validly reflect previously indicated preferences. At least in the context of the present studies, however, coin- compared to control-participants did not translate these affective reactions into decisions that reflect their initial preferences more closely.

## Supporting information

S1 Table Descriptive results for certainty, faith in intuition, superstition, belief in fate, satisfaction, and difficulty across the studies presented in this manuscript. (DOCX)
