# Supplementary material for: This outcome feels right! subjective evaluations of coin flip outcomes reflect previously stated preferences
Source: PLoS One. 2021 Jul 2;16(7):e0253751. doi: 10.1371/journal.pone.0253751 (PMC8253383; doi:10.1371/journal.pone.0253751)
Supplement: S1 Table — (DOCX) [file pone.0253751.s001.docx]

**S1 Table. Descriptive results for certainty, faith in intuition, superstition, belief in fate, satisfaction, and difficulty across the studies presented in this manuscript.**

|  |  | Certainty | | | Faith in Intuition* | Super-stition* | Belief in Fate* | Satisfaction | | | Difficulty | | |
| --- | --- | --- | --- | --- | --- | --- | --- | --- | --- | --- | --- | --- | --- |
| Study | Attract. Level | Coin | Control | Overall |  |  |  | Coin | Control | Overall | Coin | Control | Overall |
| Pretest | Medium | 5.41 (1.81) | 5.31 (1.66) | 5.36 (1.71) | - | - | - | - | - | - | - | - | - |
|  | High | 4.47 (2.24) | 4.25 (2.11) | 4.36 (2.15) | - | - | - | - | - | - | - | - | - |
|  | Low | 4.65 (2.52) | 4.69 (2.18) | 4.67 (2.33) | - | - | - | - | - | - | - | - | - |
|  | Overall | 4.84 (1.58) | 4.75 (1.34) | 4.80 (1.45) | - | - | - | - | - | - | - | - | - |
| 1 | Medium 1 | 5.11 (1.67) | 5.00 (1.59) | 5.05 (1.62) | - | - | - | - | - | - | - | - | - |
|  | Medium 2 | 4.98 (1.84) | 4.53 (1.84) | 4.75 (1.85) | - | - | - | - | - | - | - | - | - |
|  | Low 1 | 4.21 (1.96) | 4.33 (1.85) | 4.27 (1.90) |  |  |  |  |  |  |  |  |  |
|  | Low 2 | 4.00 (2.01) | 3.86 (2.07) | 3.93 (2.03) |  |  |  |  |  |  |  |  |  |
|  | High 1 | 4.77 (1.87) | 4.82 (1.77) | 4.80 (1.82) | - | - | - | - | - | - | - | - | - |
|  | High 2 | 5.23 (1.68) | 4.49 (1.95) | 4.86 (1.85) | - | - | - | - | - | - | - | - | - |
|  | Overall | 4.72 (0.94) | 4.51 (0.71) | 4.61 (0.84) | 4.57 (0.99) | 3.12 (1.36) | 3.48 (1.43) | - | - | - | - | - | - |
| 2 | Medium 1 | 4.67 (1.67) | 4.71 (1.42) | 4.68 (1.57) | - | - | - | 4.97 (1.34) | 5.10 (1.04) | 5.02 (1.23) | 4.31 (1.60) | 4.81 (1.57) | 4.49 (1.59) |
|  | Medium 2 | 4.39 (1.82) | 4.59 (1.62) | 4.45 (1.75) | - | - | - | 4.83  (1.48) | 5.00 (1.27) | 4.89 (1.41) | 4.17 (1.76) | 4.59 (1.66) | 4.30 (1.73) |
|  | Low 1 | 4.61 (1.81) | 4.55 (1.84) | 4.59 (1.81) | - | - | - | 4.97 (1.54) | 5.27 (1.35) | 5.09 (1.47) | 4.39 (1.66) | 4.18 (1.79) | 4.31 (1.70) |
|  | Low 2 | 4.64 (1.62) | 5.36 (1.22) | 4.91 (1.51) | - | - | - | 4.86 (1.36) | 5.45 (1.01) | 5.09 (1.26) | 4.47 (1.70) | 5.14 (1.39) | 4.72 (1.61) |
|  | High 1 | 4.77 (1.69) | 4.76 (2.00) | 4.77 (1.79) | - | - | - | 5.38 (1.14) | 5.10 (1.51) | 5.28 (1.28) | 5.00 (1.54) | 4.24 (2.14) | 4.73 (1.79) |
|  | High 2 | 5.47 (1.52) | 4.94 (1.43) | 5.30 (1.50) | - | - | - | 5.75 (1.02) | 5.33 (0.97) | 5.61 (1.02) | 5.31 (1.47) | 4.44 (1.58) | 5.02 (1.55) |
|  | Overall | 4.75 (0.96) | 4.87 (1.00) | 4.80 (0.97) | 4.69 (0.84) | 2.96 (1.27) | 3.27 (1.52) | 5.12 (0.89) | 5.25 (0.62) | 5.17 (0.80) | 4.60 (0.85) | 4.61 (1.07) | 4.60 (0.93) |

* Scales to assess Faith in Intuition, Superstition, and Belief in Fate were all assessed via 7-point Likert scales ranging from 1 = *do not agree* to 7 = *agree.*
